# Coronary Heart Disease and Depression or Anxiety: A Bibliometric Analysis

**DOI:** 10.3389/fpsyg.2021.669000

**Published:** 2021-06-03

**Authors:** Yan Zhou, Xue-Ping Zhu, Jing-Jing Shi, Guo-Zhen Yuan, Zi-Ang Yao, Yu-Guang Chu, Shuai Shi, Qiu-Lei Jia, Ting Chen, Yuan-Hui Hu

**Affiliations:** ^1^Department of Cardiology, Guanganmen Hospital, Chinese Academy of Traditional Chinese Medicine, Beijing, China; ^2^Clinical Medical School, Beijing University of Chinese Medicine, Beijing, China

**Keywords:** bibliometric analysis, depression, anxiety, coronary heart disease, CiteSpace

## Abstract

This study aimed to conduct a bibliometric analysis of published studies on the association between coronary heart disease (CHD) and depression or anxiety. The study also aimed to identify leading authors, institutions, and countries to determine research hotspots and obtain some hints from the speculated future frontiers. Publications about CHD and depression or anxiety between 2004 and 2020 were collected from the Web of Science Core Collection (WOSCC) database. Bibliographic information, such as authorship, country, citation frequency, and interactive visualization, was generated using VOSviewer1.6.16 and CiteSpace5.6.R5. In total, 8,073 articles were identified in the WOSCC database. The United States (2,953 publications), Duke University and Harvard University (214 publications), Psychosomatic Medicine (297 publications), and Denollet Johan. (99 publications) were the most productive country, institutions, journal, and author, respectively. The three hotspots of the research were “The relationship between depression and CHD,” “depression and myocardial infarction,” and “The characteristic of women suffering depression after MI.” The four future research frontiers are predicted to be “treating depression in CHD patients with multimorbidity,” “psychometric properties of instruments for assessing depression and anxiety in CHD patients,” “depression or anxiety in post-PCI patients,” and “other mental diseases in CHD patients.” Bibliometric analysis of the association between CHD and depressive disorders might identify new directions for future research.

## Introduction

Coronary heart disease (CHD) and depressive disorder are two major diseases that pose a great threat to public health. They have been confirmed to have a high probability to be concomitant and to have an adverse effect on each other, pushing patients to undertake heavier burdens and suffering when they are afflicted by these diseases concurrently (Whooley et al., 2008). On the one hand, patients with CHD are more likely to suffer from mental disorders because they usually endure unpleasant symptoms like angina without warning and are required to take several kinds of medications for their whole life, resulting in negative emotions like anxiety or depression (Colquhoun et al., 2013; Wu et al., 2021). To be concrete, 20–30% of patients with heart diseases are diagnosed with anxiety or depression (Larsen et al., 2016). However, the percentage of patients affected with anxiety and depression was reported to be elevated to 15–43% during the first 12 months after an acute cardiac event (Murphy et al., 2020). In addition, acute myocardial infarction (AMI) particularly correlated with depressive symptoms. The morbidity of depression in survivors of AMI is about three times more than that in common people, with 30–60% of them experiencing depressive symptoms and 15% experience major depression (Meijer et al., 2011; Myers et al., 2012; Hare et al., 2014). On the other hand, depression was determined to be a risk factor for adverse outcomes in patients with CHD with an increased risk of cardiovascular death and composite endpoint (Lahtinen et al., 2018; Wang et al., 2021). In patients with chronic coronary syndromes (CCS), those with a history of depression experienced a 2-fold rate of mortality, a higher incidence of a major adverse cardiovascular event (MACE), and a worse quality of life (QoL) at 1-year follow-up, compared with non-depressed patients (De Luca et al., 2021). Depression following AMI remains independently associated with adverse prognosis, with a 22% increase in the risk of all-cause mortality and a 13% increase in the risk of cardiovascular events (Meijer et al., 2013). Depression also shows a dose-response relationship with cardiac diseases in a recent follow-up study, the risk of 1-year MACE occurrence in patients with mild and severe depressive symptoms was 1.96 and 2.81 times that of patients without depressive symptoms, respectively (Dadkhah-Tirani et al., 2020). Compared to clinical depression, self-reported depressive symptoms are even more strongly associated with cardiac morbidity and mortality (Zuidersma et al., 2013).

Bibliometrics, first formally set up as a discipline by Alan Pritchard in 1969 (Pritchard, 1969), is receiving more attention due to the rapid development of computers and the internet. Bibliometric analysis is comprised of two basic parts: topic-centric bibliometric analysis and journal-specific studies (Muhuri et al., 2019). When applied for a journal, it concludes the overall growth structure, the quality of the publication, and the structure and citation landscape of the journal (Shukla et al., 2019). Bibliometrics is a quantitative method that applies mathematical and statistical methods to analyze scientific publications and provides a clear presentation of the distribution of contributions, hotspots, and future trends of a specific field. Although the relationship between CHD and depressive disorders gains universal attention as a hotspot of multidisciplinary research for over 10 years, few bibliometric studies related to the topic have been published, of about 1,000 reviews and over 8,000 original researches in the field. CiteSpace and VOSviewer are the commonly used bibliometric visualization tools for data analysis and visualization, and many research studies have been conducted using both the software in the medical domain. CiteSpace can conceptualize knowledge domains by generating and visualizing co-occurrence network maps of contributors and keywords, and co-citation networks of cited authors, based on bibliographical records collected from the WOSCC. VOSviewer shares similar functions in co-occurring contributions but in a more brief map (Wang et al., 2018; Gao et al., 2019).

It is significant to make bibliometric research rather than a conventional review in the highly debated topic because it can provide visualized summaries about previous publications and anticipate potential frontiers. A well-organized bibliometric study can save time for researchers by picking up frontiers for them. Hereby, we utilized the WoSCC database to collect pertinent scientific publications in the past 17 years (from 2004 to 2020), applied CiteSpace V and VOSviewer 1.6 to conduct bibliometric and visual analyses to present the global revolution and explore the hotspots and frontier directions, avid for providing researchers with useful guidance.

## Materials and Methods

Bibliometrics is a pragmatic and convenient method to quantitatively and qualitatively analyze publications of a scientific topic. It provides basic information about the contributing authors, countries, and institutions and the developing trends in a research field (Ma et al., 2020). Softwares, CiteSpace and VOSviewer, help to make bibliometric mapping a complementary methodological technique in the analysis (Cobo et al., 2011; González-Torres et al., 2020; Hernández-Torrano et al., 2020).

CiteSpace, exploited by Chaomei Chen, is a Java application that realizes the visualization of retrieved bibliographic databases. The data have to be exported from the online database in Refworks format and named as “download_^***^.txt,” which can then be discerned by CiteSpace. Knowledge about highly cited references, keywords, and contributors can be visually mapped. The keywords were then processed in CiteSpace to show the keywords with high citation bursts in the past, which were probably the most captivating aspects during a period. If certain keywords maintain a high citation burst until 2020, they are likely to be the frontiers in the future. So, the significant keywords with the strongest citation bursts are applied by researchers to summarize hotspots of specific fields in the past decades and to estimate potential research orientations in a near future. It offers convenience for researchers to have an overall understanding of the development of an academic topic and hints about potential frontiers to conduct further study. Co-cited references present a knowledge base on which previous articles rely, and the top references usually denote the most essential works in that field. In this research, CiteSpace was employed to present our knowledge on keywords, co-cited references, and speculated frontiers.

Besides, VOSviewer software which carries out analysis based on different indicators was also applied (Baier-Fuentes et al., 2020). We utilized it to present the distribution of manuscripts in terms of authors, institutions, and countries. Nodes in these maps represent the subjects, with the size of nodes indicating their publication number and the linkage between nodes showing their cooperation. In contrast to CiteSpace, VOSviewer can present terser knowledge maps with clear nodes and lines representing these basic knowledge. VOSviewer also shows the link strength of each node, which is a robust indicator of their collaborations and internationalization. Therefore, the contribution of authors, institutions, and countries was mapped by using VOSviewer in our research, and their link strength is incorporated in tables.

Data were extracted from the WoSCC database (http://apps.webofknowledge.com/) on December 18, 2020. The retrieved data were collected within 1 day to avoid any potential deviation due to daily updates. According to the WHO guideline, CHD can be divided into five types of heart diseases: silent myocardial ischemia, angina pectoris, myocardial infarction (MI), ischemic cardiomyopathy, and cardiac arrest. We, therefore, searched the database with the following search strategy: TS = (“depression” OR “depressive disorder” OR “anxiety”) AND (“coronary heart disease” OR “silent myocardial ischemia” OR “angina” OR “stenocardia” OR “myocardial infarction” OR “ischemic cardiomyopathy” OR “ischemic heart disease” OR “cardiac arrest” OR “coronary death”). Before 1991, the highest number of annual publications was 35 in 1990 and the most annual number was <10. During the 13 years from 1991 to 2003, the annual publications were maintained between 250 and 300 without a manifest change. Since 2004, the annual publications increased steadily, so the time span of the retrieved data was set from January 1, 2004 to December 18, 2020.

**Figure 1:**
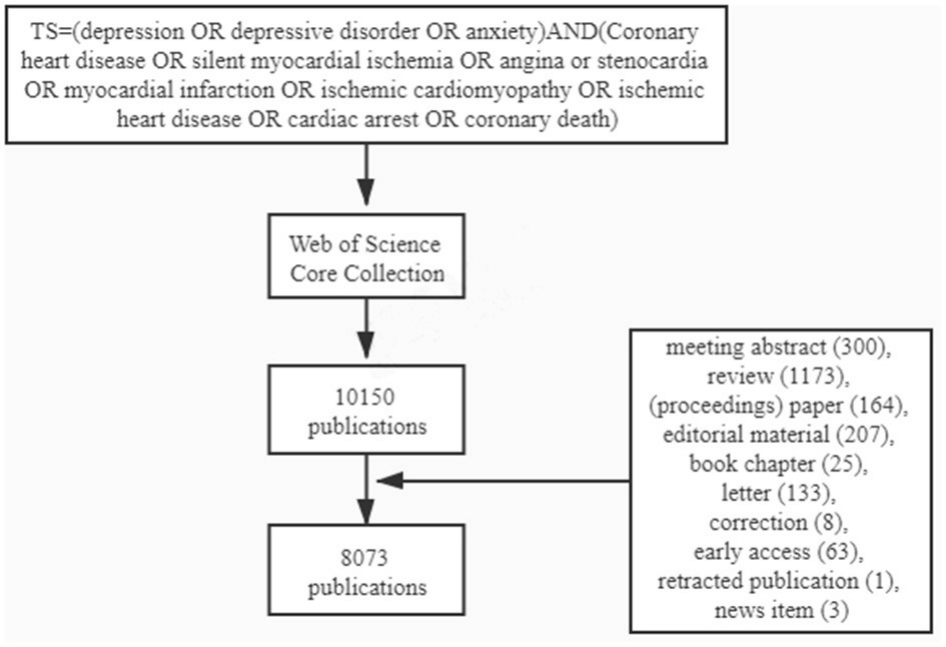
Flow chart of the inclusion criteria.

The search strategy is shown in Figure 1. About, 10,150 publications were obtained, and the following documents were excluded: meeting abstract (300), review (1,173), proceedings paper (164), editorial material (207), book chapter (25), letter (133), correction (8), early access (63), the retracted publication (1), and news item (3). Then, the information of the remaining 8,073 documents which were mostly comprised of original articles and meta-analysis was downloaded and was analyzed with VOSviewer 1.6.16 to identify top countries, institutions, and authors, and using CiteSpace 5.6.R5 to determine keywords, co-cited references, and burst citations. The information in tables and figures was made with a full counting system.

## Results

### Distribution of Annual Publication

**Figure 2:**
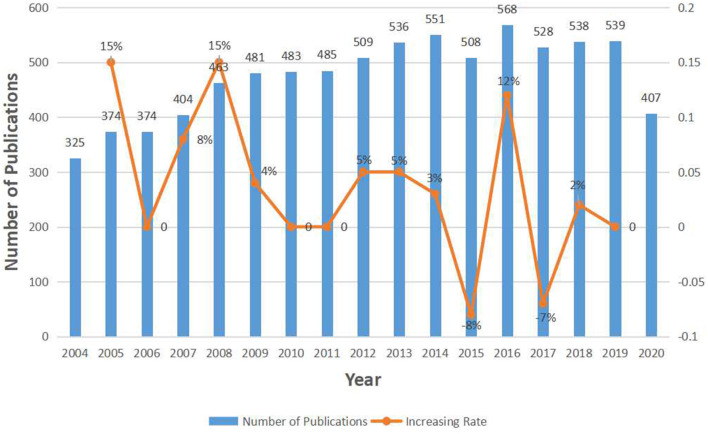
The number of annual publications relating to research about CHD and depression or anxiety from 2004 to 2020.

A total of 8,073 articles about depression or anxiety with CHD were retrieved from 2004 to December 18, 2020. The annual publication could elucidate the trend and significance of an academic topic, so we present the number of annual publications and increasing rate in the form of a histogram (Figure 2). For these 17 years, annual article productions rise steadily, which indicates that related topic captures increasingly more attention from researchers. From 2004 to 2014, the annual publication number elevated nearly 70% from 325 to 551. Then the number dropped slightly in 2015 but reached its peak in 2016 with a total of 568 publications. The production had a second subtle drop in 2017 and kept nearly flat in the following 2 years. It can be inferred that the publications in 2020 would decrease by about 20% compared to last year.

### Distribution of Countries and Institutions

**Figure 3:**
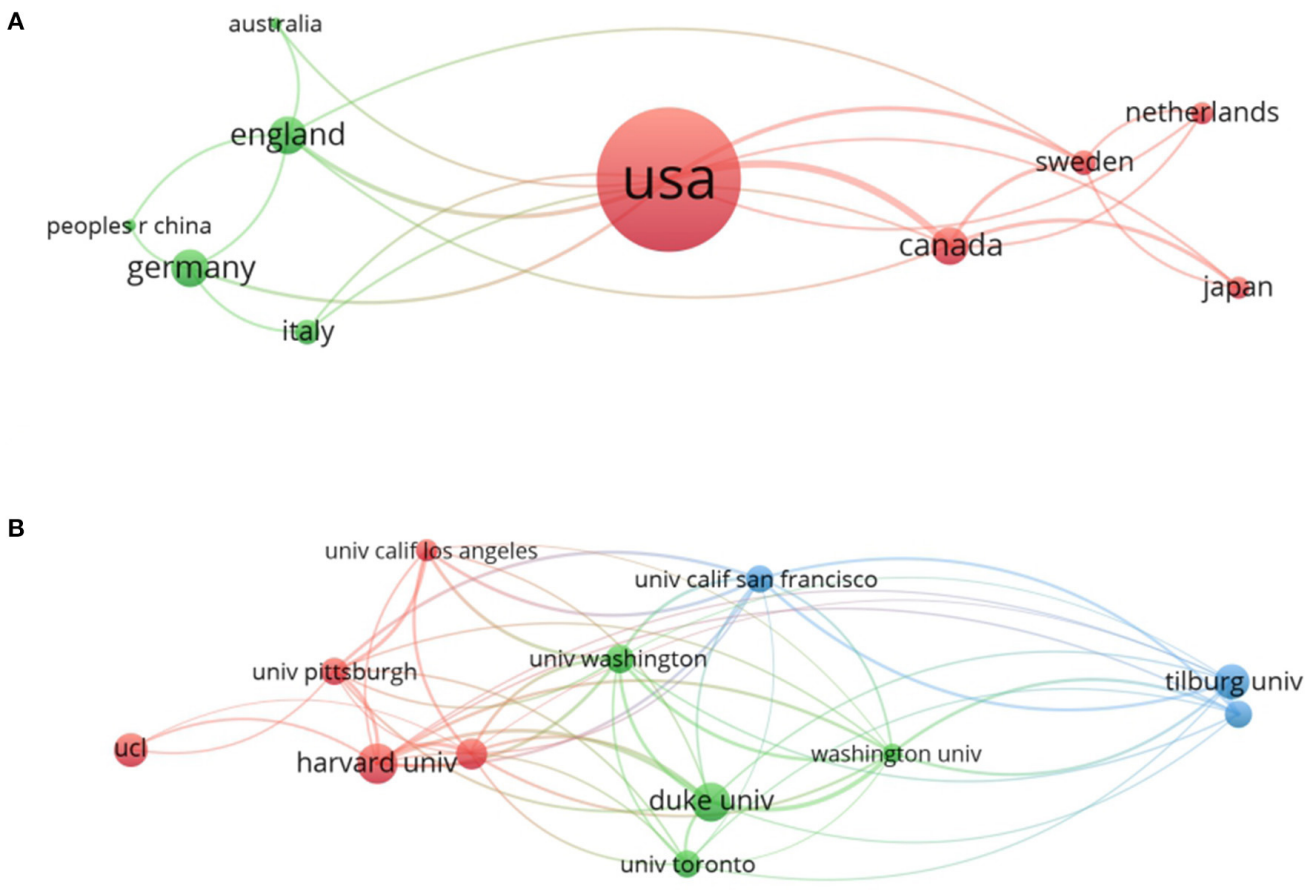
The analysis of related countries and institutions. **(A)** Network of countries/territories engaged in the research. **(B)** Network of institutions engaged in the research. In both networks, the larger the node is, the more contribution the country or institution has made to that field. The link strength between two nodes represents their collaborations and the wider the linking is, the more cooperation is between them. The linking strength between the nations and the US is listed as follows: Australia (109), England (114), Canada (221), Germany (104), Sweden (69), and Japan (47). The linking strength between institutes and Duke University are listed as follows: Harvard University (15), Tilburg University (3), Columbia University (8), the University of Washington (24), the University of California, San Francisco (4), the University of Toronto (9), Pittsburg University (6), and Groningen University (2).

To evaluate contributions made by different countries and institutions and to figure out underlying cooperative relationships between them, we applied VOSviewer to show the co-occurrence maps of countries and institutions in Figure 3. In addition, we listed the top 10 countries and institutions which enjoyed the most publications and their total link strength in Table 1. Researchers from about 100 countries/territories contributed to the 8,073 articles and the United States (US), England, Germany, Canada, and The Netherlands ranked the top five productive countries. The US published 2,953 articles, which is more than the other countries, making it the most critical country in the research of CHD with depression or anxiety. Among the top 10 institutions, six of them belong to the US, which can explain its large proportion of total publications. The value of total link strength demonstrates the collaboration of a subject with others. As shown in Table 1, the US presents a much higher total link strength than other countries, with about 56% increase than England, which is the second top country. Therefore, the US cooperated the most with other countries and contributed the most in this field. And as the two most productive institutions, Duke University showed a slight increase in total link strength than Harvard University, which indicated it preferred a collaborative mode than the latter one. There are also certain universities like Sweden and the University of Pittsburgh showing correspondingly higher total link strength even though their contribution is less. So, the value was not related completely to the contribution, but less preeminent countries or institutions may present an intense cooperative intention.

**Table 1:** Top 10 countries and institutions contributed to the publications.

**Rank**	**Country**	**Frequency/ Total link strength**	**Institution**	**Frequency/ Total link strength**
1	USA	2,953/1,611	Duke University	214/565
2	England	763/1,035	Harvard University	214/529
3	Germany	599/596	Tilburg University	185/367
4	Canada	588/653	University College London	179/391
5	Netherlands	575/598	Columbia University	167/412
6	Australia	481/521	University of Washington	152/368
7	China	466/297	University of California San Francisco	149/406
8	Sweden	362/479	University of Toronto	148/454
9	Italy	332/365	University of Pittsburgh	143/457
10	Japan	248/160	University of Groningen	138/288

### Distribution of Journals and Authors

**Figure 4:**
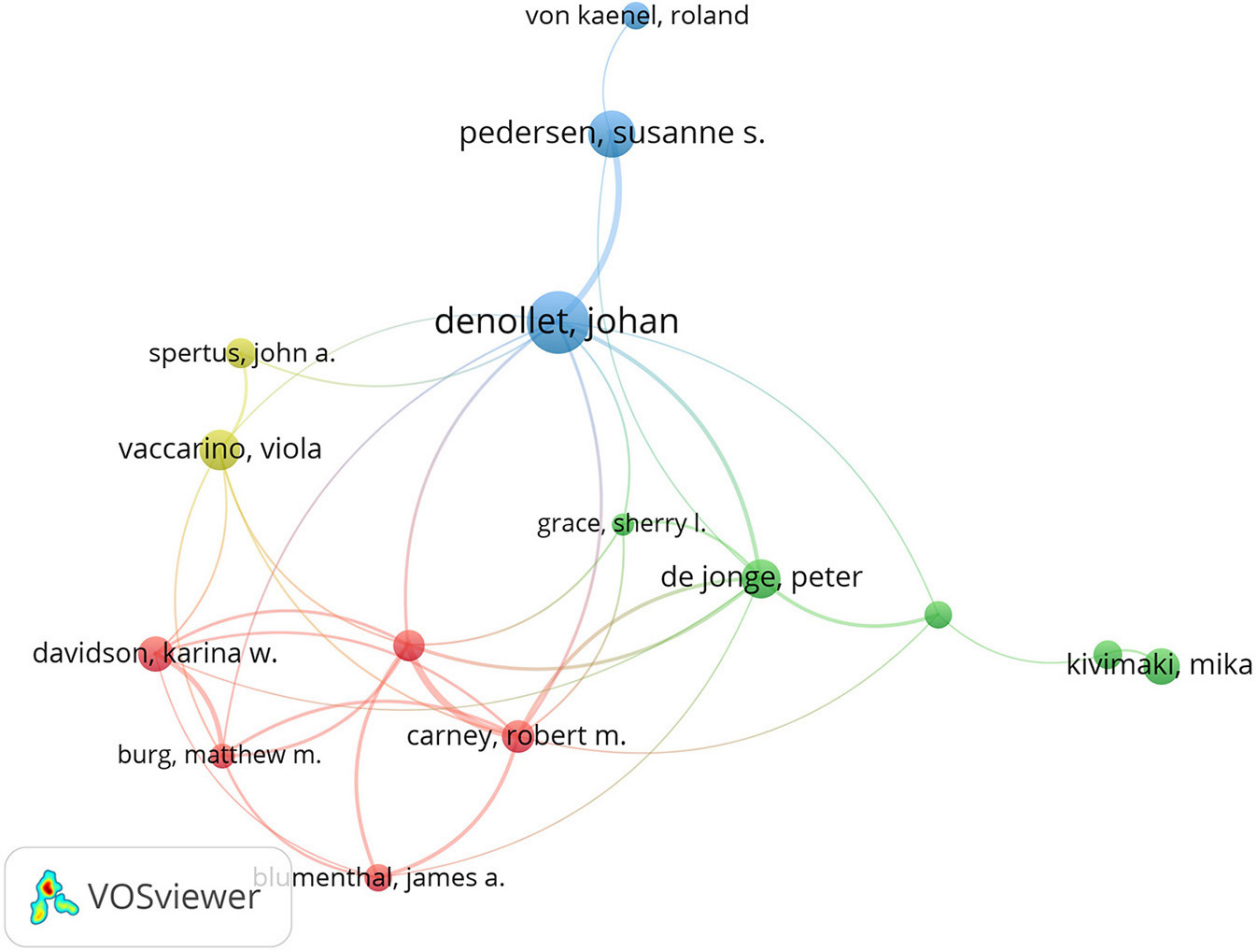
The network of authors contributed to the research about CHD and depression or anxiety from 2004 to 2020. The linking strength between authors and Denollet J. is listed as follows: Pedersen S. S. (30), De Jonge P. (11), Vaccarino V. (1), Carney R. M. (5), and Freedland K. E. (5).

About 1,005 journals were published with a total of 8,073 articles. Table 2 lists the top 10 journals and six of them are from the US. Psychosomatic Medicine had the highest number of articles of 297 (3.679%), followed by the Journal of Psychosomatic Research which published 196 papers (2.428%), and the American Journal of Cardiology which published 178 articles (2.205%). Over 34,000 authors contributed to a total of 8,073 publications, and we applied VOSviewer to create the co-authorship map to find potential collaborations among different authors. Figure 4 presents the network of authors, and the top 10 productive authors and their total link strength are listed in Table 3. In the network of authors, the largest node was Denollet and Johan (99 articles), who mainly focused on Type D personality and its adverse effect on cardiac prognosis (Denollet, 2005). As shown in Figure 4 and Table 3, Denollet and Johan hold strong links with as many as the top 10 authors with a total citation burst of 90, from which we can infer that a large research group includes Denollet, Johan, Carney, Robert M., De jonge, Peter and Pedersen (Pedersen and Denollet, 2004), and Susanne S. By contrast, the total citation burst of Steptoe A. was much lower than the others, which can be inferred that the researcher may prefer an independent study.

**Table 2:** Top 10 journals published most articles on the research.

**Rank**	**Journal**	**Frequency (%) *N* = 8,073**	**IF 2019**	**Country affiliation**
1	Psychosomatic Medicine	297 (3.679%)	3.702	United States
2	Journal of Psychosomatic Research	196 (2.428%)	2.86	England
3	American Journal of Cardiology	178 (2.205%)	2.57	United States
4	PLoS ONE	166 (2.056%)	2.74	United States
5	International Journal of Cardiology	152 (1.883%)	3.229	Ireland
6	Journal of Affective Disorders	135 (1.672%)	3.892	Netherlands
7	American Heart Journal	114 (1.412%)	4.153	United States
8	BMJ Open	81 (1.003%)	2.496	England
9	Journal of Electrocardiology	75 (0.929%)	0.944	United States
10	Journal of Cardiovascular Nursing	72 (0.892%)	1.675	United States

**Table 3:** Top 10 active authors in the research.

**Rank**	**Author**	**Frequency (%) *N* = 8,073**	**Total link strength**
1	Denollet J.	99 (1.226%)	90
2	Pedersen S. S.	74 (0.917%)	63
3	Kivimaki M.	74 (0.917%)	59
4	De Jonge P.	68 (0.842%)	73
5	Vaccarino V.	61 (0.756%)	51
6	Von Kanel R.	60 (0.743%)	26
7	Carney R. M.	56 (0.694%)	86
8	Freedland K. E.	55 (0.681%)	84
9	Davidson K. W.	53 (0.657%)	88
10	Steptoe A.	52 (0.644%)	5

### Distribution of Co-cited References and Top-Cited Articles

**Figure 5:**
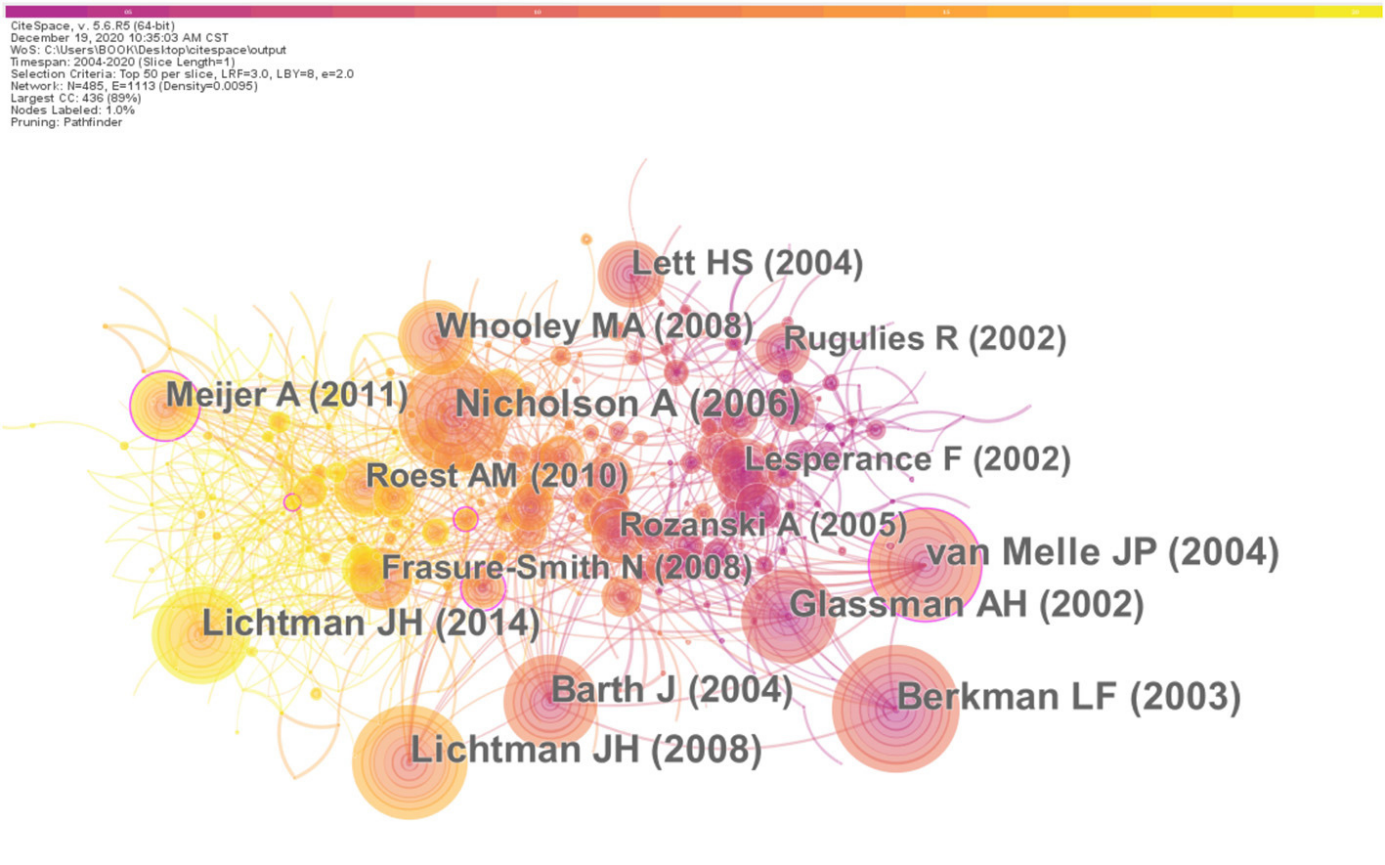
The analysis of Co-citation network of references from publications on the research about CHD and depression or anxiety from 2004 to 2020. The centrality accounts for the significance of the reference. The centrality and counting of each top co-cited references are listed as follows with the format of “author name (centrality, counting) and the order conform to the rank by counting: Berkman L. F. (0.03, 259), Leichtman J. H. (0.05, 231), Van Melle J. P. (0.11, 225), Nicholson A. (0.05, 223), Leichtman J. H. (0.07, 205), Barth J. (0.08, 199), Glassman A. H. (0.02, 192), Whooley M. A. (0.08, 157), Meijer A. (0.11, 142), and Lett H. S. (0.05, 140).

CiteSpace was then applied to analyze and visualize co-cited references of the 8,073 articles with a time span ranging from 2004 to 2020 and a time slice of 1 year. The network of co-cited references on the research is consisting of references with higher centrality and citation counts is presented in Figure 5, which is convenient to determine the crucial knowledge base in the field. The main information about the top 10 highest co-cited references is summarized in Table 4. The most c0-cited reference was written by Berkman L. F., who conducted a randomized trial from 1996 to 2001 and concluded that the cognitive behavior therapy did not increase event-free survival in depression and psychosocial intervention group but the intervention did improve depression and social isolation, compared with the depression and usual care group (Berkman et al., 2003). The second and the fifth articles were both recommendations made by the American Heart Association (AHA) for providing patients with CHD with healthcare for the assessment, referral, and treatment of depression in 2008 to elevating depression as a risk factor in patients with the acute coronary syndrome (ACS) in 2014 (Lichtman et al., 2008, 2014). Four articles including the third, the fourth, the sixth, and the ninth were meta-analyses. The third and the ninth articles calculated the increased risk of adverse cardiovascular outcomes in patients with post-MI (van Melle et al., 2004).

**Table 4:** Top 10 co-cited references (CR) in the research.

**Rank**	**Frequency**	**Author**	**Year**	**Source**	**Co-cited reference**
1	259	Berkman L. F.	2003	JAMA	Cognitive behavior therapy improved depression and social isolation but did not increase event-free survival.
2	231	Leichtman J. H.	2008	Circulation	The evidence linking depression with CHD and provides recommendations for healthcare.
3	225	Van Melle J. P.	2004	Psychosomatic Medicine	Post-MI depression is associated with a 2- to 2.5-fold increased risk of impaired cardiovascular outcome.
4	223	Nicholson A.	2006	European Heart Journal	Depression has not yet to be established as an independent risk factor for CHD.
5	205	Leichtman J. H.	2014	Circulation	The American Heart Association should elevate depression as a risk factor for adverse medical outcomes in ACS patients.
6	199	Barth J.	2004	Psychosomatic Medicine	Depression has to be considered a relevant risk factor in patients with CHD.
7	192	Glassman A. H.	2002	JAMA	Sertraline is a safe and effective treatment for recurrent depression in patients with recent MI or unstable angina.
8	157	Whooley M. A.	2008	JAMA	The association between depressive symptoms and adverse cardiovascular events was largely explained by behavioral factors, particularly physical inactivity.
9	142	Meijer A.	2011	General Hospital Psychiatry	Post-MI depression is associated with a 1.6- to 2.7-fold increased risk of impaired outcomes within 24 months.
10	140	Lett H. S.	2004	Psychosomatic Medicine	Evidence for a relationship between depression and adverse clinical outcomes is substantial but randomized clinical trials are needed.

Articles with high citations show the hotspots in the field of interest with the citation number revealing their significance, so the top 10 cited articles were summarized in Table 5. The second and fourth articles were both related to the heavy burden posed by depressive disorder as it contributes to many other diseases including cardiovascular diseases (CVDs). The fifth and the eighth articles concluded that ischemic heart disease as a key disease accounting for years of life lost (YLLs) due to premature mortality in the US, while depressive disorder as an important factor for years lived with disability (YLDs) in China.

**Table 5:** Top 10 cited articles in the research.

**Rank**	**Frequency**	**Author**	**Year**	**Source**	**Main points**
1	2,386	Dowlati Y.	2003	Biological Psychiatry	Depression is accompanied by activation of the Inflammatory responsive system (IRS).
2	2,135	Moussavi S.	2008	Lancet	The urgency of addressing depression as a public-health priority is to reduce disease burden and disability, and to improve the overall health of populations.
3	1,609	O'Donnell M. J.	2004	Lancet	Ten risk factors including depression are associated with 90% of the risk of stroke.
4	1,503	Hankey G. J.	2006	PLoS Medicine	Depressive disorder is a leading cause of burden. Major depressive disorder contributes to suicide and ischemic heart disease which emphasize the importance of including depressive disorders as a public-health priority.
5	1,424	Murray C. J. L.	2014	JAMA-Journal of the American Medical Association	Ischemic heart disease was one key disease accounting for years of life lost due to premature mortality (YLLs), while major depressive disorder and anxiety disorder were for years lived with disability (YLDs) in the United States in 2010.
6	1,369	Chen Z. M.	2004	Lancet	In a wide range of patients with acute MI, adding clopidogrel 75 mg daily to aspirin and other standard treatments safely reduces mortality.
7	1,333	Rosengren A.	2002	Lancet	Psychosocial stressors is associated with increased risk of acute myocardial infarction, suggesting that approaches aimed at modifying these factors should be developed.
8	1,100	Yang G. H.	2008	Lancet	Ischemic heart disease was one leading cause of death in China. Mental and behavioral disorders, substance use disorders, and musculoskeletal disorders were responsible for almost half of all YLDs.
9	1,369	Roux A. V. D.	2011	Biology of Disadvantage: Socioeconomic Status and Health	Features of neighborhoods or residential environments may affect diseases including CHD and depressive disorders.
10	1,094	De Hert M.	2004	World Psychiatry	Cardiovascular diseases are more prevalent among people with severe mental illness (SMI).

### Distribution of Research Areas

**Figure 6:**
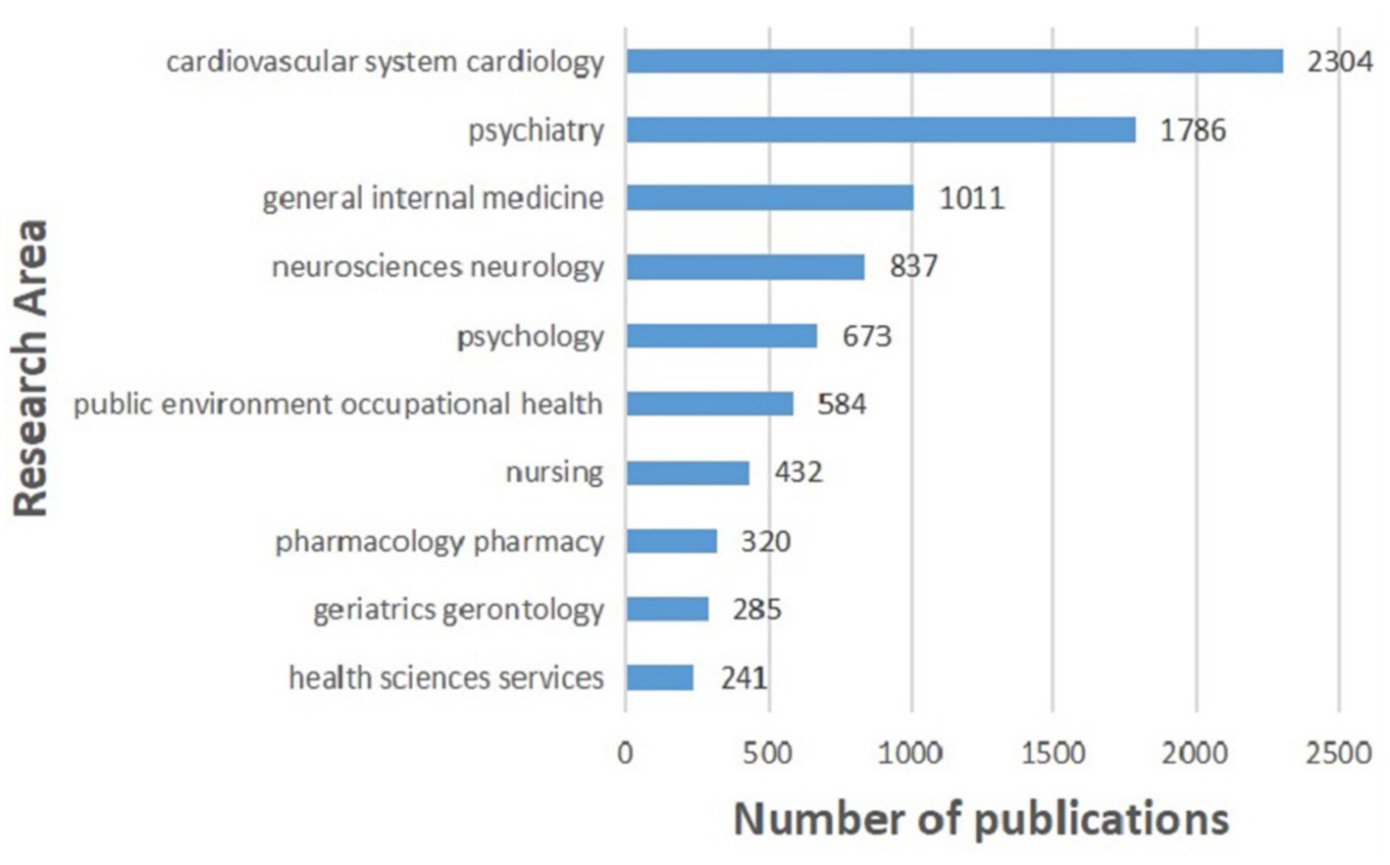
Top 10 research areas relating to the research about CHD and depression or anxiety from 2004 to 2020.

Figure 6 shows the top 10 research areas and the publication numbers in each area. Cardiology, psychiatry, and general internal medicine are the top three most studied fields.

### Distribution of Keywords

**Figure 7:**
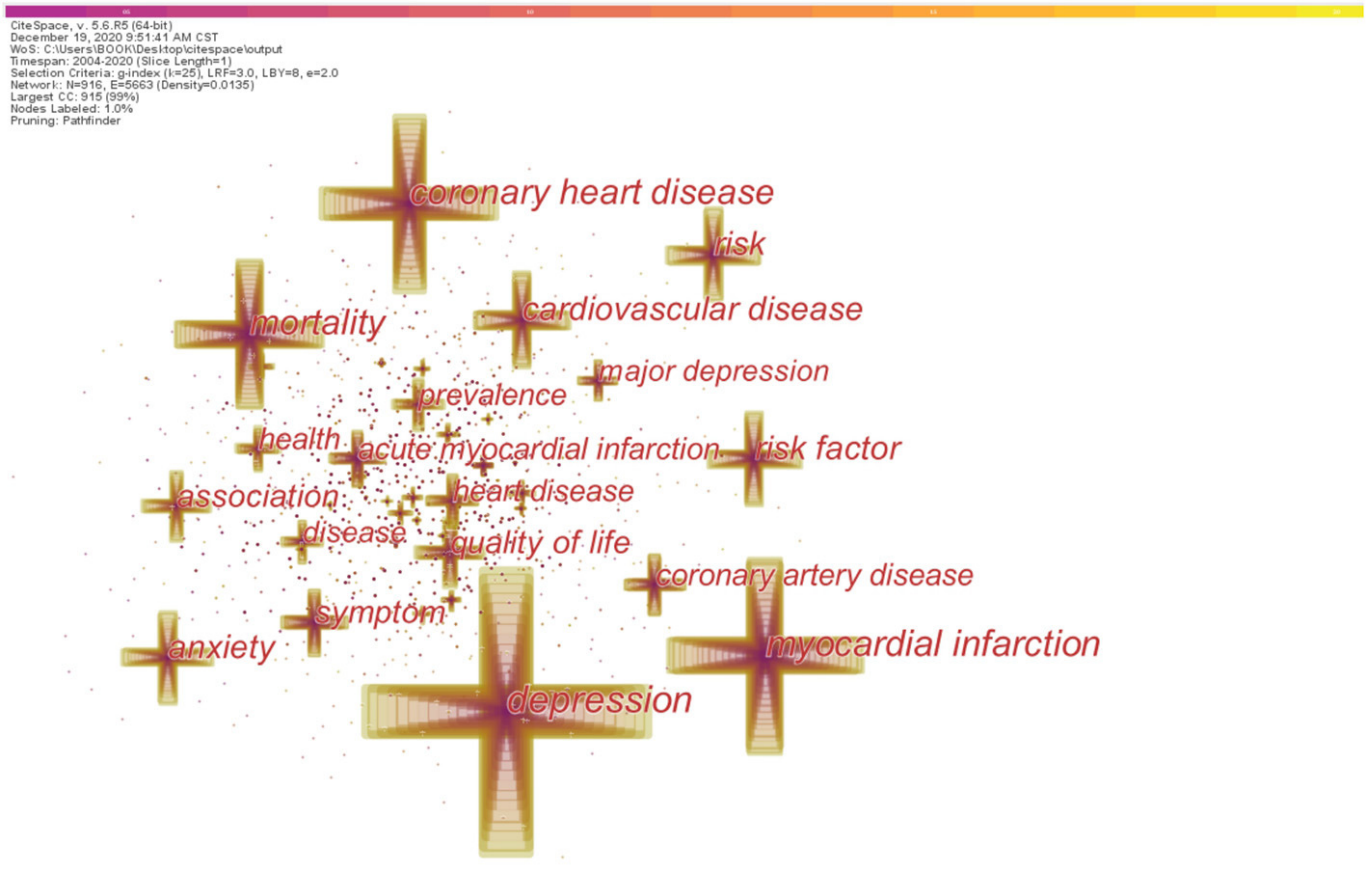
The analysis of keywords relating to the research about CHD and depression or anxiety from 2004 to 2020. The larger the cross is, the higher frequency each keyword has. The frequency of the top keywords is listed in Table 6.

To acquire hotspots and frontiers of the retracted publications from 2004 to 2020, keywords and their citation bursts have to be analyzed. CiteSpace was applied to set up a keyword knowledge co-occurrence map in Figure 7. The larger the node is, the more significant the keyword will be. To get a more concrete impression on keywords, we listed the 20 most frequent keywords with their frequencies in Table 6. The top 20 keywords were “depression,” “myocardial infarction,” “coronary heart disease,” “mortality,” “risk factor,” “risk,” “cardiovascular disease,” “anxiety,” “quality of life,” “association,” “symptom,” “coronary artery disease,” “heart disease,” “acute myocardial infarction,” “prevalence,” “health,” “major depression,” “disease,” “meta analysis,” and “women.”

**Table 6:** Top 20 keywords in terms of frequency in the research.

**Rank**	**Keyword**	**Frequency**
1	Depression	3,374
2	Myocardial infarction	2,377
3	Coronary heart disease	2,233
4	Mortality	1,843
5	Risk factor	1,266
6	Risk	1,260
7	Cardiovascular disease	1,231
8	Anxiety	1,220
9	Quality of life	981
10	Association	974
11	Symptom	925
12	Coronary artery disease	847
13	Heart disease	828
14	Acute myocardial infarction	824
15	Prevalence	763
16	Health	733
17	Major depression	664
18	Disease	656
19	Meta analysis	552
20	Women	499

Keywords were then analyzed in CiteSpace to view the words with high citation bursts, which can show the frontiers in a given period. In Table 7, we presented the top 40 keywords with the highest citation bursts that last until 2020 to obtain a basic understanding of the frontiers in recent years and speculate future frontiers. The top 10 keywords were “multimorbidity,” “recommendation,” “psychometric property,” “global burden,” “percutaneous coronary intervention (PCI),” “scientific statement,” “all-cause,” “European society,” “trend,” and “perceived stress.”

**Table 7:** Top 40 keywords with the strongest citation bursts and last until 2020.

**Keyword**	**Strength**	**Begin**
Health behavior	5.4577	2014
Sex	4.5086	2014
Abuse	3.9593	2014
Escitalopram	4.3407	2015
Scientific statement	8.7745	2015
Perceived stress	6.0501	2015
PHQ9	3.9319	2015
Frailty	5.1422	2015
All cause	8.2136	2015
Recommendation	9.9486	2016
Country	4.7401	2016
Trend	6.4885	2016
Atrial fibrillation	4.3624	2016
Medical comorbidity	3.7528	2016
Multimorbidity	10.9419	2016
Global burden	9.1181	2016
National health	3.9413	2017
Arthritis	4.2342	2017
Resilience	4.3793	2017
Outcome	4.8551	2017
European society	7.7055	2017
Psychosocial stress	4.9032	2017
Receptor	3.9504	2018
Psychosis	3.9035	2018
PTSD	5.3848	2018
Acute ischemic stroke	4.4892	2018
Veteran	4.6596	2018
Clinical feature	3.9035	2018
Elevation MI	5.5716	2018
Lipid	3.9035	2018
Bipolar disorder	5.631	2018
COPD	4.0422	2018
Brain	4.5532	2018
Psychometric property	9.1973	2018
Cardiac rehabilitation	3.867	2018
Adolescent	3.5592	2018
PCI	8.9488	2018
Insurance research database	5.053	2018

### Hotspots of CHD With Depression or Anxiety

Based on the keywords with the most frequencies, we summarized research hotspots on CHD with depression or anxiety in the following aspects:

(1) The relationship between depression and CHD

Although several pieces of evidence showed the link between depression and deterioration of CHD, many authors argued that there was no convincing evidence proving depression as an independent causal risk factor for CHD (Blumenfield et al., 2012). In 2014, the AHA declared a recommendation to elevate depression to the status of a risk factor for adverse medical outcomes in patients with ACS (Lichtman et al., 2014). A prospective longitudinal study published in 2016 concluded that the strength of association between depression and CHD incidence was of a greater magnitude than any typical and atypical risk factors (O'Neil et al., 2016).

(2) Depression and MI

Researchers tend to pay more attention to MI than other types of CHD in pertinent studies. It has been widely accepted that MI can increase the risk of depression while depression can also enhance the risk of getting MI. Both major and minor depression post-MI are associated with higher mortality and new cardiovascular events (van Melle et al., 2004). Depression will also have a negative influence on the QoL in patients post-MI (de Jonge et al., 2006). The underlying mechanism between depressive disorder and MI may depend on enhanced immune activity, stimulated hypothalamic-pituitary-adrenal (HPA) axis, decreased polyunsaturated fatty acids, increased platelet activity and serotonin, and negative health behavior and personal characteristics like type D personality (Kuyper and Honig, 2008). Even if depression seems to be much less critical in a patient with MI, physicians are supposed to make an early diagnosis of depression and offer positive treatments. Treatments including fluoxetine, sertraline, cognitive behavioral therapy, mirtazapine, and citalopram have been tested by researchers on depressed patients with MI, and all these patients showed the therapeutic effect to some extent (Roose et al., 1998; Glassman et al., 2002; Berkman et al., 2003; Honig et al., 2007; Lesperance et al., 2007).

(3) The characteristic of women suffering depression after MI

With a higher lifetime risk for depression and anxiety, women are the area of interest for researchers to test whether sexuality matters in the outcome of depressed patients with CHD. It turned out that women are more likely to develop anxiety and depression after MI than men and get worsened outcomes (Taylor, 2015). A study supports that depression is an independent risk factor for the incidence of CHD in women, which could be explained from etiological, behavioral, and treatment perspectives (O'Neil et al., 2016). Evidence indicates that endothelial dysfunction and lower flow reserve in a microvascular system, due to lower resting coronary flow, lower possibility in identifying CHD risk factors, lower rate of attending cardiac rehabilitation, and so on, may account for the susceptibility of women to adverse CHD outcomes (Mosca et al., 2010; Kobayashi et al., 2015). Specific recommendations have been carried out for women by AHA, including “Effectiveness based Guidelines for the Prevention of Cardiovascular Disease in Women−2011 Update,” which classifies CVD risk classification for women (Mosca et al., 2011).

### Frontiers in the Future

Based on the keywords with the highest citation bursts that last till 2020, we forecast frontiers as follows:

(1) Treating depression in patients with CHD with multimorbidity

Multimorbidity, is defined as concurrently having at least two chronic conditions, is more common, especially, in the elderly as it rises with the aging of the population (Salive, 2013). Multimorbidity is followed by deteriorated life quality, higher medical cost, and, at last, an earlier death compared to people without multimorbidity (Rillamas-Sun et al., 2016). CVDs can develop into systemic failure due to multiple connections to organismal metabolism, which in turn is associated with multimorbidity (Cruz-Avila et al., 2020). Depression is frequently concurrent with multiple chronic diseases, and a study concluded that depression is central in patterns of multimorbidity and is associated with the incidence of some common chronic diseases, including ischemic heart disease, justifying that screening and treating depression in those who are at risk for developing chronic disease are appropriate (Birk et al., 2019). The author called for future research to investigate the roles of health behaviors in the association between depression and multimorbidity. Also, a recent study emphasized the role of physical activity (PA) and concluded that people with more than one chronic condition and whose status of PA was inactive had higher odds of elevated depressive symptoms, while only in men, engaging in weekly recommended PA targets may attenuate the association of heart disease with depressive symptoms (Andrade-Lima et al., 2020). It can be predicted that increased health behaviors will be considered and taken into a clinical trial to determine their influence on multimorbidity, depression, and CHD, which will further conduce to a better QoL in certain patients as rightful and positive interventions.

(2) Psychometric properties of instruments for assessing depression and anxiety in patients with CHD

In as early as 2004, researchers investigated the psychometric properties of the Hospital Anxiety and Depression Scale (HADS) as a screening instrument for patients with ACS and confirmed that the HADS was useful in assessing symptoms of psychological distress in patients with ACS (Martin et al., 2004). Psychometric properties of assorted instruments have been studied in cardiac patients using the Depression in the Medically Ill-18 (DMI-18), the Depression in the Medically Ill-10 (DMI-10), the MacNew Heart Disease HRQL questionnaire (MacNew), the German HeartQoL questionnaire, and the Patient Health Questionnaire (PHQ-9) (Hilton et al., 2006; Yu et al., 2008; Gholizadeh et al., 2019; Huber et al., 2020). It can be inferred that the psychometric properties of more instruments will be tested on patients with CHD to determine their validity and reliability of assessing depression and anxiety in a specific group of patients.

(3) Depression or anxiety in patients with post-PCI

Depression or anxiety could have a mutual effect with PCI in patients with CHD. From one perspective, to see if depression and anxiety could predict adverse cardiovascular events for patients with post-PCI, a recent prospective study concluded that depression is associated with an increased risk for MACEs post-PCI, independent of anxiety; while anxiety is also associated with MACEs, it has no additional predictive value when co-occurring with depression (Hou et al., 2020). A meta-analysis also concluded that depressive symptoms were associated with MACEs independently (Song et al., 2020). From another perspective, to figure out if PCI could affect depression and anxiety positively, a study with 153 recruited subjects determined the prevalence of depression and anxiety in patients undergoing PCI for ACS was only 10.5 and 7.5%, respectively, which bolstered that PCI could not only improve angina and rehospitalization but also decrease the incidence of depression and anxiety in patients with ACS (Mujtaba et al., 2020).

Some studies also import potential beneficial interventions in patients with post-PCI and concluded that interventions, such as comprehensive cardiac rehabilitation management and the 24-Form Tai Chi not only ameliorated depressive symptoms but were also conducive to better QoL (Liu et al., 2020; Wang et al., 2020). We predict that further studies will discover and prove more salutary interventions for patients with post-PCI, which could provide helpful guidelines for recovery strategies.

(4) Other mental diseases in patients with CHD

Post-traumatic stress disorder (PTSD) has been widely known to have a link with increased risk for CVD (Edmondson and von Kanel, 2017). Acute cardiac events can also lead to PTSD. A study showed that black and younger patients were more vulnerable to PTSD after MI (Rocha et al., 2008). Besides, whether improvement in the condition of PTSD will be beneficial for outcomes in patients with CHD remains unknown. In a 2–7 year follow-up study, clinically meaningful PTSD improvement failed to show association with the incidence of CVD after stratifying by depression status (Scherrer et al., 2020). Further research is needed using longer follow-up to decipher the puzzle. In men and women diagnosed with bipolar disorder (BPD), CVD contributes 17.4 and 22.0%, respectively, reduction in overall life expectancy (Nielsen et al., 2020). However, the vascular-bipolar link is difficult to study due to the latency between the onset of BPD, which is usually from an early age, and subsequent vascular disease. Therefore, studies often focus on risk factors for vascular disease, but further research should focus on identifying novel treatment approaches and relevant mediators of this relationship, including lifestyle, medications, and systemic biological mediators (Goldstein et al., 2020).

## Discussions

This research applied VosViewer and CiteSpace software to conduct a bibliometric analysis of published articles relating to studies about CHD and depression or anxiety. The annual publication presents an overall escalating trend in pertinent studies. In addition to the basic knowledge about the research topic, we also generalized the research hotspots in the past years and speculated frontiers in the near future. Filling research gaps in the field is the task for future researchers, and the gaps were presented in each frontier in the results. In the first frontier, treating depression in patients with CHD with multimorbidity, the research gap is the potential salutary roles of some health behaviors in the association between depression and multimorbidity. As for the second frontier, psychometric properties of instruments for assessing depression and anxiety in patients with CHD, the research gap is the psychometric properties of some instruments being tested on patients with CHD to determine their validity and reliability of assessing depression and anxiety in a specific group of patients. As for the third frontier, depression or anxiety in patients with post-PCI, the research gap is to discover and prove more salutary interventions in patients with post-PCI, which could provide helpful guidelines for recovery strategies. As for the fourth frontier, other mental diseases in patients with CHD, the research gap is whether improvements in the condition of PTSD will be beneficial for the outcomes in patients with CHD, and further research is required using longer follow-up studies to fill the gap. In addition, future research studies about BPD and CHD should focus on identifying novel treatments and relevant mediators of their relationship, including lifestyle, medications, and systemic biological mediators. These research gaps can guide future researchers to decipher the puzzles and improve their cooperation. Health policies and guidelines can be supplemented in the future. For instance, after some other PAs like Tai-chi has been proven to improve the health condition of patients with CHD and depression, these salutary PAs may be included in health policy and guidelines.

Overall, the analysis could benefit pertinent researchers who are interested in the scientific topic by offering them a vivid background of the topic and potential research targets in the future. Because the topic is related to both CVD and mental disorders, the target researchers are mainly comprised of cardiologists and psychologists. Due to the existing research gaps, the cooperation between them may be increased.

However, there are some limitations in the study. The bibliometric analysis retrieved publications solely from the WOSCC database, which implies that some influential documents may have been neglected in this analysis. Therefore, future studies can complement this bibliometric analysis with other databases, such as Scopus, EBSCO, and Procuest. In addition, the WOSCC database contains several indexes, including Emerging Sources Citation Index (ESCI), which complements the highly selective indexes by providing earlier visibility for sources under evaluation as a part of the rigorous journal selection process. So, the retrieved data may contain such manuscripts which are under evaluation. We excluded reviews in the study to primarily present the bibliometric characteristics of original articles, while the exclusion may conceal some hotspots in the field. Also, the time span was set from the year 2004 due to the annual publication number, but some pertinent manuscripts published before 2004 also presented high value in the field (Dusseldorp et al., 1999; Gorman and Sloan, 2000; Scheubel et al., 2003). Further, the total recruited manuscripts may contain certain weak and peripheral ones, which can distort the analysis to some extent.

## Conclusions

Based on the WOSCC database, bibliometric tools were applied to analyze articles related to CHD and depressive disorders from 2004 to 2020 to determine publication patterns and contributors and to identify recent trends in research in this field. The results may help investigators to determine the status of research topics and to identify new directions for future research in this field. Overall, annual productivity is rising steadily over the past 17 years and it can be predicted that the trend will continue to rise. The findings showed that most publications were from the US, and the most productive institution and author in terms of the number of publications and total link strength were Duke University and Denollet J., respectively. The three hotspots of the research were “The relationship between depression and CHD,” “Depression and myocardial infarction,” and “The characteristic of women suffering depression after MI.” The top four research frontiers are “treating depression in CHD patients with multimorbidity,” “psychometric properties of instruments for assessing depression and anxiety in CHD patients,” “depression or anxiety in post-PCI patients,” and “other mental diseases in CHD patients.”

## Data Availability Statement

The raw data supporting the conclusions of this article will be made available by the authors, without undue reservation.

## Author Contributions

YZ and X-PZ designed the study, retrieved the data, performed the statistical analysis, and wrote the first draft. YZ made the further modifications. J-JS, G-ZY, Z-AY, and Y-GC processed the figures. SS, Q-LJ, and TC processed the tables. Y-HH supervised the whole process and provided modification advice. All authors contributed to the article and approved the submitted version.

## Conflict of Interest

The authors declare that the research was conducted in the absence of any commercial or financial relationships that could be construed as a potential conflict of interest.
